# Comparison of CT number calibration techniques for CBCT-based dose calculation

**DOI:** 10.1007/s00066-015-0890-7

**Published:** 2015-09-24

**Authors:** Alex Dunlop, Dualta McQuaid, Simeon Nill, Julia Murray, Gavin Poludniowski, Vibeke N. Hansen, Shreerang Bhide, Christopher Nutting, Kevin Harrington, Kate Newbold, Uwe Oelfke

**Affiliations:** 1 Joint Department of Physics, Institute of Cancer Research, The Royal Marsden NHS Foundation Trust, London, SM2 5NG UK; 2 The Royal Marsden Hospital, Sutton, Surrey, SM2 5PT Downs Road UK; 3 The Institute of Cancer Research, London, SM2 5NG UK; 4 Department of Medical Physics, Karolinska University Hospital, Stockholm, 171 76 Sweden

**Keywords:** Cone beam computed tomography, Adaptive radiation therapy, ART, Dose calculation, Hounsfield units, Density, Cone-Beam-CT, Adaptive Strahlentherapie, Dosisberechnung, Hounsfield-Einheiten, Dichte

## Abstract

**Purpose:**

The aim of this work was to compare and validate various computed tomography (CT) number calibration techniques with respect to cone beam CT (CBCT) dose calculation accuracy.

**Methods:**

CBCT dose calculation accuracy was assessed for pelvic, lung, and head and neck (H&N) treatment sites for two approaches: (1) physics-based scatter correction methods (CBCT_r_); (2) density override approaches including assigning water density to the entire CBCT (W), assignment of either water or bone density (WB), and assignment of either water or lung density (WL). Methods for CBCT density assignment within a commercially available treatment planning system (RS_auto_), where CBCT voxels are binned into six density levels, were assessed and validated. Dose-difference maps and dose-volume statistics were used to compare the CBCT dose distributions with the ground truth of a planning CT acquired the same day as the CBCT.

**Results:**

For pelvic cases, all CTN calibration methods resulted in average dose-volume deviations below 1.5 %. RS_auto_ provided larger than average errors for pelvic treatments for patients with large amounts of adipose tissue. For H&N cases, all CTN calibration methods resulted in average dose-volume differences below 1.0 % with CBCT_r_ (0.5 %) and RS_auto_ (0.6 %) performing best. For lung cases, WL and RS_auto_ methods generated dose distributions most similar to the ground truth.

**Conclusion:**

The RS_auto_ density override approach is an attractive option for CTN adjustments for a variety of anatomical sites. RS_auto_ methods were validated, resulting in dose calculations that were consistent with those calculated on diagnostic-quality CT images, for CBCT images acquired of the lung, for patients receiving pelvic RT in cases without excess adipose tissue, and for H&N cases.

## Abstract (de)

**Ziel:**

Ziel dieser Arbeit ist der Vergleich und die Validierung mehrerer CT-Kalibrierungsmethoden zur Dosisberechnung auf der Grundlage von Kegelstrahlcomputertomographie(CBCT)-Aufnahmen.

**Methoden:**

Bei 4 Becken-, 3 Lungen- und 4 Kopf-Hals-Patienten wurde die Genauigkeit der Dosisberechnung auf der Basis von CBCT-Aufnahmen für die folgenden Ansätze untersucht: einerseits Rekonstruktion der CBCT-Aufnahmen mithilfe eines Streukorrekturalgorithmus (CBCT_r_) und andererseits 3 verschiedene Methoden zur expliziten Zuweisung der Elektronendichten auf Basis des CBCT-Datensatzes (W: Zuordnung von Wasserdichte auf dem gesamten CBCT, WB: Zuordnung von entweder Wasser- oder Knochendichte, WL: Zuordnung von entweder Wasser- oder Lungendichte) sowie der in einem kommerziellen Planungssystem implementierten Methode der automatischen Zuordnung von 6 Dichtestufen auf Basis der CBCT-Hounsfield-Werte (RS_auto_). Als Grundlage zur Evaluierung der Methoden diente die Dosisverteilung, welche anhand einer am gleichen Tag wie die CBCT aufgenommenen Planungs-CT berechnet wurde. Die Genauigkeit der einzelnen Ansätze wurde anhand von Dosis-Volumen-Statistiken und lokalen Dosisunterschieden beurteilt.

**Ergebnisse:**

Bei den Datensätzen mit Tumoren im Beckenbereich ist die mittlere Dosisabweichung für alle Kalibrierungsmethoden unter 1,5 %, wobei RS_auto_ in einer überdurchschnittlichen Abweichung für Patienten mit einem höheren Anteil von Fettgewebe resultiert. Die mittlere Abweichung für Kopf-Hals-Patienten beträgt unter 1,0 %, wobei CBCT_r_ (0,5 %) und RS_auto_ (0,6 %) am besten abschneiden. WL und RS_auto_ resultieren für die Patienten mit Lungentumoren in Dosisverteilungen, welche der Referenzdosisverteilung entsprechen.

**Schlussfolgerung:**

RS_auto_ zur Kalibrierung von CBCT-Aufnahmen zur Dosisberechnung ist eine aussichtsreiche Methode für die untersuchten Indikationen. Es wurde gezeigt, dass mithilfe von RS_auto_ die Dosisberechnung auf der Basis von CBCT-Aufnahmen von Lungen- und Kopf-Hals-Erkrankungen sowie für Tumoren im Beckenbereich bei Patienten ohne ein Übermaß an Fettgewebe in einer akkuraten Dosisverteilung resultiert.

Since its introduction, cone beam CT (CBCT) has been mainly utilized for image-guided radiotherapy (IGRT) [1–4]. CBCT images generated prior to treatment can also be used for adaptive radiotherapy (ART) [5, 6] in various ways, ranging from using the CBCT to estimate the dosimetric effect of patient weight loss, CBCT assessment in order to choose a “plan-of-the-day,” to dose recalculation and treatment plan generation on CBCT images [7–11]. Commercially available treatment planning systems (TPSs) are now able to implement ART strategies.

CBCT image quality is dependent on acquisition parameters and, compared with diagnostic-quality planning CT images, exhibit increased artifacts and poorer image contrast owing to increased scatter, which itself is dependent on the size of the scanned object/patient [12–14]. CBCT images (CBCTs) generated using XVI on Elekta treatment units are not in true CT numbers (CTNs) and, therefore, in their original form, such CBCTs cannot be used directly for accurate dose computation [12]. In order to be able to perform CBCT-based ART, it is important that dose can be calculated accurately (i.e., consistent with that calculated on diagnostic CT images) on the CBCT image; various methods have been suggested for CTN adjustment in order to achieve this [12, 15–21].

Strategies for CTN adjustment include calibration of the CBCT image voxel values with physical density following CBCT acquisitions of phantoms with inserts of differing densities [15–19]. Richter et al. [17] proposed a method based on average CBCT values for separate treatment sites in order to generate population-specific conversion curves for brain, head and neck (H&N), thorax, and prostate treatment sites. Apart from taking a large amount of time to define, conversion curves are subject to errors caused by image artifacts and patient variability, resulting in differences of 5 % and larger between doses calculated on CBCTs compared with CT-based calculation [12, 17]. Patient-specific conversion curves have also been investigated [20]. However, these methods are still prone to dosimetric errors resulting from CBCT artifacts.

Poludniowski et al. have shown that differences, between the doses on CBCT and CT, of less than 2.5 % can be achieved when the CBCT has been reconstructed after scatter correction of the individual projections [20, 22]. The implementation of such an approach can be slow and difficult to introduce into a clinical workflow. Commercially available systems that generate CBCTs in CTNs by using sophisticated scatter-correction algorithms are starting to become commercially available in recent software releases. However, the accuracy of dose calculation on such images has yet to be validated.

Relatively unsophisticated methods, such as density overrides of regions of interest, can be used to populate CBCTs with density values that result in acceptable dose calculation accuracy [12, 20, 21]. Such strategies can be fast to implement and can easily be adapted into clinical workflows. These techniques are not as sensitive to the problems, such as image artifacts, that the more sophisticated methods struggle to cope with.

Unlike other studies presented for density assignment methods [12, 21] and other approaches [15–20, 23], we use radiotherapy planning CTs (PCT_CBCT_) acquired on the same day as the CBCT as the ground truth for dose calculation. Thus, we have selected patients with minimal anatomical differences between CBCTs and planning CTs, eliminating the need for, and uncertainties associated with, deformable image registration (DIR) [24–27]. Using the original planning CT (PCT_orig_), which may have been captured weeks before the CBCT, as the ground truth for dose calculation is unsuitable in the context of ART because establishing an estimate of the difference in dose between that calculated on the PCT_orig_ and CBCT is itself a subject of importance. We have investigated three anatomical sites: H&N, pelvis (prostate and bladder), and lung.

The aim of this work was to assess the CBCT dose calculation accuracy for density override approaches and to compare this with the physics-based scatter correction of Poludniowski [20, 22]. We evaluated the accuracy of the RayStation TPS (V3.99, RaySearch Laboratories, Stockholm, Sweden) density override approach and compared it with more simple density override methods investigated in the literature [12]. We aimed to establish, for different anatomical locations, the methods that were best in terms of CBCT dose calculation accuracy. The methods for CTN adjustment investigated in this work are transferable to dose calculation on magnetic resonance (MR) images and could, therefore, be of interest to MR-based ART [28].

## Patients and methods

We selected patients who had a CBCT and diagnostic CT (both with the same immobilization and/or support devices) on the same day. The study consisted of four H&N cases, four pelvis cases (two prostate and two bladder treatment sites), and three lung cases (two left upper lobe GTVs, one right upper lobe GTV). The RayStation TPS was used for dose calculations, using a collapsed cone algorithm. The median time difference between the CBCT and PCT_CBCT_ was 59 min (range: 17–215 min).

### CBCT dose calculation

**Fig. 1:**
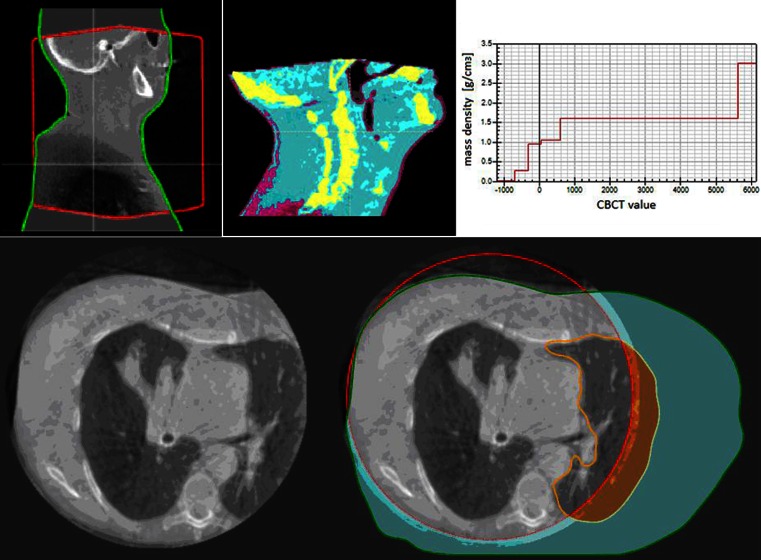
The RayStation treatment planning system (TPS) cone beam computed tomography (CBCT) CT number (CTN) adjustment method. *Top row from left*: Sagittal slice of a CBCT image of an H&N cancer patient viewed within the TPS; the CBCT after density assignment by the TPS (regions assigned as bone are shown as *yellow*, for example); CTN-density table generated for the CBCT image. *Bottom row, left:* Typical CBCT acquisition of a patient with a tumor in their right lung and (*right*) the same CBCT image but with the field of view (*red contour*), external (*green contour*), and left lung (*orange contour*) regions of interest displayed

The CBCTs were acquired with Elekta XVI-V4.5 (XVI Elekta, Stockholm, Sweden); the scanning parameters are displayed in Table 1. To calculate dose on the CBCT images, an external patient contour was defined. First, an external contour of the CBCT image was defined using the TPS automated algorithm that uses a thresholding technique based on the image data. Small holes were then removed from the threshold-generated volume and finally contours not associated with the largest object were deleted. Next, a field-of-view (FOV) region of interest (ROI) was defined for the CBCT image set (see Table 1 for FOV parameters). Finally, with a priori knowledge of the planning CT scan, an automated algorithm (available in the TPS) updated the external contour of the CBCT (where necessary) to extend beyond the CBCT FOV. A missing tissue ROI (with density = 1 g/cm^3^) is created and is defined as the newly generated external ROI exterior to the FOV ROI. Figure 1 (top left) displays the FOV, external, and missing tissue ROIs for an H&N CBCT image as a red contour, green contour, and gray color wash, respectively.

**Table 1:** CBCT scanning parameters for the three anatomical sites investigated in this study^a^

Protocol name	KeV	mA	mS	FOV (cm)	Scan length (cm)	Filter	Nominal dose CTDI (mGy)
Chest S20	120	25	40	26	26	F0 (none)	9.00
Head and neck S20	100	10	10	26	26	F0 (none)	0.45
Pelvis M10	120	100	40	40	13	F1 (bowtie)	27.00

*CTDI* computed tomography dose index.

^a^The H&N and lung patients were scanned with a field of view (FOV) of 26 cm allowing a partial gantry rotation for acquisition, but limiting the full patient contour (i.e., missing the shoulders on the H&N patients and only seeing the treated sides of the lung patients), whereas acquisitions for the pelvis patients were made with the 40-cm FOV.

### CTN adjustment using CBCT reconstruction

Following Poludniowski [20, 22, 29], CBCT reconstructions (CBCT_r_) were made after scatter correction. The software developed in-house explicitly simulates and corrects for scatter in the projection images using the patients PCT_orig_ as a prior [20, 22]. A patient-independent look-up table was applied to the reconstructed images in order to convert the reconstructed CTNs into physical density [29].

### CTN adjustment using density overrides

The “water only” (W) method, where all tissue was assigned as water (1 g/cm^3^), was investigated in all three treatment sites. For pelvis and lung cases, “water-and-bone” (WB) and “water-and-lung” (WL) techniques were also investigated, respectively. For WB, the CBCT voxels were assigned as either water or bone. Bone ROIs were generated for each CBCT image using a segmentation algorithm based on gray value thresholds on the PCT_orig_ data and copying the resulting ROIs onto the CBCT image. The bone ROIs were edited if necessary, to ensure they were appropriate with reference to the CBCT image. The PCT_orig_ bone ROIs were additionally used to establish an appropriate density to assign to the CBCT bone ROI. The average CTN of bone ROIs on PCT_orig_ was 1,400, which was similar to previously reported data [30] and equivalent to 1.35 g/cm^3^. This density was used for all cases included in this study. In the WL technique, the patient was assumed to be made up of either water or lung tissue (0.2 g/cm^3^), where the density of lung was calculated using similar methods as those used to determine the pelvic bone density.

More sophisticated density override techniques were investigated using the CBCT density-assignment tools available in the TPS where six different set densities (air, lung, adipose tissue, connective tissue, cartilage/bone, and higher density for prosthesis) are assigned to the CBCT image. This is achieved by binning the CBCT image histogram into six density levels. The CBCT image thresholds are individual to each patient whereas the assigned density values are fixed (Fig. 1). An automatic algorithm (RS_auto_) extracts the various density thresholds by approximating the CBCT image histogram with two normal distributions which were then interrogated to determine the threshold values. The physical densities applied by the TPS to the various tissue types were: air = 0.00121 g/cm^3^, lung = 0.26 g/cm^3^, adipose = 0.95 g/cm^3^, connective tissue = 1.05 g/cm^3^, cartilage/bone = 1.6 g/cm^3^, and other (such as prosthetic hip) = 3 g/cm^3^. The consideration of the other override approaches (W, WL, WB) provides a useful benchmark and understanding for the more complex RS_auto_ methodology. Table 2 outlines the CTN adjustment techniques investigated in this study.

**Table 2:** The different CTN adjustment methods investigated in this study

Method name	Method description	Sites used in study
CBCT_r_	CBCT reconstruction after scatter correction Poludniowski	Pelvis, H&N, lung
W	All tissue was assumed to be water (1 g/cm^3^)	Pelvis, H&N, lung
WB	CBCT voxels assigned as either water or bone	Pelvis
WL	CBCT voxels assigned as either water or lung	Lung
RS_auto_	CBCT voxels automatically binned into six density levels	Pelvis, H&N, lung

*CBCT*_*r*_ physics-based scatter correction methods, *W* assignment of water density to the entire CBCT, *WB* assignment of either water or bone density, *WL* assignment of either water or lung density, *RS*_*auto*_ RayStation TPS, *H&N* head and neck.

### Limited FOV for lung CBCTs

At our institution, the FOV for CBCT acquisitions for lung patients does not encompass the entire patient contour (Table 1, Fig. 1). When performing CTN adjustment using CBCT_r_ or RS_auto_, it may, therefore, be necessary to perform an additional density override to the contralateral lung. As well as applying a density override of 1 g/cm^3^ to the missing tissue ROI (region shaded turquoise in Fig. 1), an additional density override of 0.2 g/cm^3^ was applied to the left lung outside of the FOV (shaded orange in Fig. 1). If CBCT scans of the lung were to be routinely used for dose calculation, we would recommend using either a medium or large FOV to ensure the entire patient contour at the superior/inferior extent of the tumor was encompassed. This contralateral lung density issue has only a small effect on treatments where all beams enter ipsilaterally. However, for treatments where one (or more) beams entered contralaterally, the lung ROI from the planning CT scan was copied to the CBCT image and a density override was applied (Fig. 1).

### Data analysis

Dose statistics and dose-difference maps were generated using the TPS. Dose–volume histogram (DVH) analysis was performed on ROIs that were propagated to the CBCT from the PCT_CBCT_ scan.

## Results

**Fig. 2:**
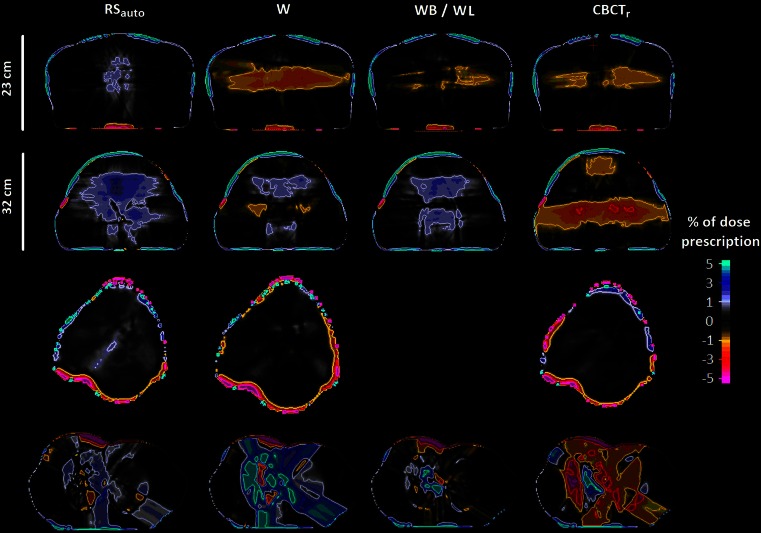
Dose difference maps for between doses calculated on CBCT images and the ground truth (PCTCBCT). From *top row* to *bottom*: (1) a pelvic case with anterior-posterior distance (D_AP_) = 23 cm; (2) a pelvic case with D_AP_ = 32 cm; (3) an H&N case; and (4) a lung case. All dose difference maps are presented as a percentage of the prescribed dose

Differences in dose-volume statistics between that calculated on the CBCT and the ground truth, for all three treatment sites, are shown in Table 3 and Fig. 2.

**Table 3:** Dose difference statistics of target and organs at risk as calculated on the CBCT compared with the ground truth

		CBCTr mean%diff (% range)	W mean%diff (% range)	WB mean%diff (% range)	WL mean%diff (% range)	RS_auto_ mean%diff (% range)
*Pelvic treatments*						
**CTV**	D_median_	0.8 (0.5–1.2)	0.3 (− 1.3–1.7)	− 1.0 (− 1.6–− 0.1)		− 1.3 (− 2.6–− 0.1)
	D_95 %_	0.8 (0.0–2.0)	0.0 (− 2.4–1.4)	− 1.5 (− 2.7–− 0.4)		− 1.5 (− 3.7–− 0.2)
	D_98 %_	0.8 (0.0–2.0)	− 0.1 (− 2.6–1.4)	− 1.5 (− 2.8–− 0.4)		− 1.7 (− 3.9–− 0.4)
	D_2 %_	1.2 (0.7–1.5)	0.7 (− 0.2–1.9)	− 0.7 (− 1.4–− 0.2)		− 0.8 (− 1.2–0.1)
**Femoral heads**	D_50 %_	2.4 (1.6–3.1)	0.9 (− 1.2–3.9)	− 1.1 (− 1.8–− 0.5)		− 0.7 (− 1.5–− 0.1)
**Rectum**	D_50 %_	1.4 (− 0.2–4.2)	0.2 (− 1.1–1.1)	− 1.0 (− 1.8–0.5)		0.7 (− 1.3–0.6)
**Bladder**	D_mean_	0.4 (0.0–0.8)	0.1 (− 0.3–0.4)	− 1.0 (− 1.2–− 0.8)		− 1.5 (− 1.6–− 1.4)
**Average** ^**a**^		1.4 (− 0.3–4.2)	0.0 (− 2.6–3.9)	− 1.0 (− 2.8–0.5)		− 1.1 (− 3.9–2.0)
**Absolute average** ^**a**^		1.4 (0.0–4.2)	0.7 (0.0–3.9)	1.0 (0.1–2.8)		1.2 (0.0–3.9)
*Head and neck treatments*						
**CTV**	D_median_	0.3 (− 0.1–1.0)	0.4 (0.0–1.3)			− 0.1 (− 0.6–0.1)
	D_95 %_	0.5 (0.1–0.9)	1.9 (0.2–4.7)			0.2 (− 0.3–1.0)
	D_98 %_	0.4 (0.0–0.9)	0.4 (0.0–0.8)			− 0.1 (− 0.3–0.0)
	D_2 %_	0.3 (− 0.2–1.0)	0.8 (0.1–1.4)			− 0.1 (− 0.4–0.2)
**Spinal cord**	D_2 %_	0.7 (0.0–1.7)	1.4 (1.0–2.5)			0.5 (− 0.1–1.0)
**Brain stem**	D_2 %_	0.5 (− 0.6–1.4)	0.5 (− 1.7–1.6)			− 0.5 (− 2.5–0.6)
**Parotids**	D_mean_	0.5 (0.0–1.1)	0.4 (− 0.5–1.4)			− 0.3 (1.5 − 0.4)
**Average** ^**b**^		0.4 (− 0.6–1.7)	0.8 (− 1.7–4.7)			− 0.1 (− 2.5–1.0)
**Absolute average** ^**b**^		0.5 (0.0–1.7)	1.0 (0.0–4.7)			0.6 (0.1–2.5)
*Lung treatments*						
**CTV**	D_median_	2.4 (1.9–2.9)	− 6.8 (− 7.7–− 6.1)		0.4 (− 0.6–0.9)	− 1.3 (− 2.1–− 0.8)
	D_95 %_	0.0 (− 1.7–1.7)	− 7.0 (− 7.4–− 6.5)		0.5 (− 2.0–1.8)	− 1.1 (− 1.7–− 0.7)
	D_98 %_	− 1.3 (− 4.8–2.2)	− 6.9 (− 7.1–− 6.6)		0.3 (− 2.7–2.2)	− 1.0 (− 1.5–− 0.7)
	D_2 %_	3.0 (1.9–4.1)	− 6.2 (− 6.6–− 5.9)		0.8 (− 0.1–1.4)	− 0.9 (− 2.3–0.5)
**Heart**	D_mean_	− 0.2 (− 3.4–2.9)	− 3.4 (− 8.7–1.7)		− 0.9 (− 3.3–1.2)	− 2.2 (− 4.0–− 0.8)
**Lungs**	D_mean_	2.9 (− 1.0–8.0)	− 7.8 (− 12.7–4.6)		0.4 (− 1.6–1.6)	− 1.8 (− 3.9–− 0.3)
**Spinal cord**	D_2 %_	3.7 (3.0–4.4)	− 2.4 (− 5.7–1.4)		1.1 (0.0–1.7)	− 0.8 (− 1.9–0.5)
**Average** ^**c**^		1.5 (− 4.8–8.0)	− 5.9 (− 12.7–1.7)		0.2 (− 3.3–1.8)	− 1.4 (− 4.0–0.5)
**Absolute average** ^**c**^		2.8 (1.0–8.0)	6.1 (1.4–12.7)		1.3 (0.0–3.3)	1.4 (0.3–4.0)

*W* water, *WB* water or bone, *WL* water or lung, *CTV* clinical target volume.

^a^Average values are the mean of the dose difference statistics for all CTVs (D_98 %_, D_95 %_, mean, D_50 %_, D_ 2 %_), D_50 %_ for femoral heads, D_mean_ for bladder, D_50 %_ and D_2 %_ for rectum.

^b^The average values are the mean of the dose difference statistics for all CTVs (D_95 %_, mean, D_50 %_, D_ 2 %_), D_50 %_ for parotids, D_2 %_ for brain stem and spinal cord.

^c^The average values are the mean of the dose difference statistics for the CTV (D_98 %_, D_95 %_, mean, D_50 %_, D_ 2 %_), mean dose to heart and healthy lung, D_2 %_ for spinal cord.

**Fig. 3:**
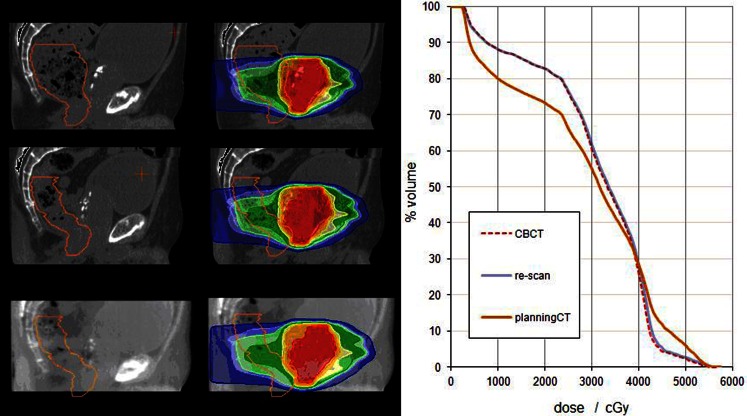
*Left*: Sagittal images of a prostate RT treatment plan with dose calculated on (*top*) the PCT_orig_; (*middle*) PCT_CBCT_; and (*bottom*) the CBCT. The rectum is shown as an *orange contour* and the dose is shown in color wash relative to the prescribed dose. *Right:* dose–volume histogram of the rectum for the dose calculated on the PCT_orig_ scan (*orange line*); the PCT_CBCT_ (*blue line*); and the CBCT (*red dashed line*). The RS_auto_ method was used for CTN adjustment of the CBCT image. The rectum OAR was similar in size and shape on the CBCT and the PCT_CBCT_ but was very different to that seen on the PCT_orig_ scan. *PCT* planning computed tomography, *CBCT* cone beam CT, *CTN* CT number, *OAR* organ at risk

Internal anatomy variability of a prostate case is illustrated in Fig. 3, demonstrating that the use of PCT_CBCT_ as the ground truth is, in some instances, essential for meaningful DVH dosimetric comparison for CBCT dose calculation accuracy. Previous studies [12, 15–21, 23] of CBCT dose calculation accuracy have either used rigid (which may have been acquired weeks prior to the CBCT and therefore exhibit substantial anatomical differences) or deformable image registrations (and their associated uncertainty) between the original planning CT and CBCT.

### Pelvis cases

**Fig. 4:**
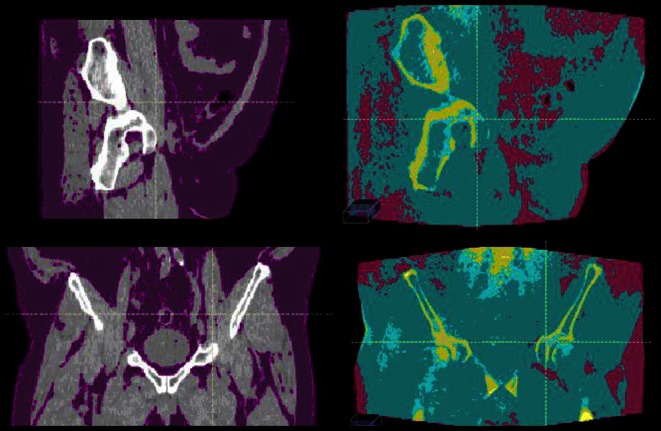
Sagittal (*top row*) and coronal slices (*bottom row*) of a pelvic case with a high proportion of adipose tissue. *From left to right*: the PCT_CBCT_ with tissue density < 0.95 g/cm^3^ colored *purple,* and CBCT with RS_auto_ CTN adjustment. In the CBCT images *purple*, *turquoise*, and *yellow* represent adipose (0.95 g/cm^3^), connective tissue (1.05 g/cm^3^), and bone (1.6 g/cm^3^), respectively. *PCT* planning computed tomography, *CBCT* cone beam CT, *CTN* CT number

RS_auto_ tended to underestimate the proportion of lower-density tissue present in larger patients (Fig. 4), corresponding to an underestimation of the dose. This underestimation has been observed in a larger cohort of pelvic cases that were not used in this dose validation study as they did not have a CBCT and CT acquired on the same day. However, for patients with anterior-posterior distance (D_AP_) < 25 cm, RS_auto_ resulted in the most accurate dose distributions of all CTN adjustment methods with an average of 0.7 % absolute difference in dose when compared with the ground truth. Furthermore, for patients with D_AP_ < 25 cm, the WB method worked well with the assignment of water (1 g/cm^3^) to all non-bone tissue, being adequate in such cases as these patients commonly have similar amounts of lower-density (0.95 g/cm^3^) and connective (1.05 g/cm^3^) tissue. However, for patients with higher proportions of adipose tissue, the WB method did not perform as well. The W method performed better than WB for patients with D_AP_ > 25 cm, with the additional adipose tissue being balanced out by the higher-density bone. The CBCT_r_ method was unable to accurately reconstruct higher-density material for the larger patient, resulting in a similar effect to that observed with the W method for patients with D_AP_ < 25 cm. For this treatment site, the RS_auto_ method produced the best results when compared with the ground truth, with an average absolute difference of 0.7 % (range: 0.1–2.5 %) when only considering patients with D_AP_ < 25 cm. For CBCT images acquired around the pelvis of patients with D_AP_ < 25 cm, we therefore recommend using the RS_auto_ method for CTN adjustment. However, the WB and W methods worked with sufficient accuracy [20] for patients with D_AP_ < 25 cm and > 25 cm, respectively.

### H&N cases

All three methods investigated for CTN adjustment for H&N cases resulted in acceptable [20] average absolute dose deviations of ≤ 1.0 % when compared with the ground truth, with CBCT_r_ (0.5 %) and RS_auto_ (0.6 %) performing best. For CBCT images acquired of the H&N, on the basis of the results presented in Table 3, its integration within a TPS, and its ease and speed of implementation, we recommend using the RS_auto_ method for CTN adjustment. Larger dose differences were observed in the inferior part of the target at the level of the shoulders. Similar to the methods employed for the contralateral lung ROIs (Fig. 1), this dose disagreement may be improved by generating additional bone ROIs for the shoulders to enable more accurate dose calculation on the CBCT data set.

### Lung cases

Assuming that density overrides external to the FOV ROI were applied as per Fig. 1, the WL and RS_auto_ methods generated dose distributions most similar to PCT_CBCT_ calculations, with average absolute dose differences of 1.3 and 1.4 %, respectively (Table 3). When the RS_auto_ or CBCT_r_ methods were applied to VMAT treatments without applying additional contralateral lung density overrides, the average absolute dose difference increased to over 3.0 %. The W method performed poorly for lung cases as, unlike the other methods investigated for this site, it did not account for the lower-density lung tissue present. On the basis of the results presented in Table 3, for CBCT images acquired of the lung, we recommend using either the RS_auto_ or WL method for CTN adjustment. This study did not investigate the dependence of CBCT-based dose calculations on lung intra-fraction motion.

## Discussion

For the sites investigated in this study, the differences between the doses calculated on the CBCT compared with the diagnostic CT, when using RS_auto_, were similar or better than other reported results. Fotina et al. demonstrated that density override techniques applied to CBCT scans of the prostate resulted in dosimetric differences below 2 %, whereas the use of conversion curves to calculate dose on CBCT images has been shown to result in differences of up to 5 % [12]. However, Richter et al. demonstrated that conversion curves specific to anatomical sites can result in comparable dose calculation accuracy with differences below 2 % [17].

Owing to weight loss during radiotherapy for H&N cancer [7, 24, 31, 32], for the H&N cases it was necessary to use the PCT_CBCT_ as the input to the algorithm within the TPS used to define the CBCT body contour. When the PCT_orig_ was used, the large differences in the body contour between the PCT_CBCT_ and CBCT meant dose differences between that calculated on the CBCT and the ground truth could not be assessed with confidence. The use of the PCT_CBCT_ data as a priori knowledge for this validation study reduced the uncertainty of the shape of the CBCT body contour, resulting in more accurate estimations of CBCT dose. If CBCT scans of the H&N were to be routinely used for dose calculation, we would recommend using either a medium or large FOV to ensure the entire patient contour at the superior/inferior extent of the tumor was imaged in order to generate the entire patient contour on the CBCT image. Cupping artifacts can have a large effect on CBCT dose calculations, and the degree to which they affect the dose depends on the CTN adjustment method used. CBCT-density look-up table techniques are the most sensitive in this context. However, CBCT dose distributions generated using density override methods, such as those discussed in this study, are also affected by image quality with increasing sensitivity with increasing number of density bins. However, the results indicate that binning the CBCT voxels into six densities is an appropriate methodology that results in dose distributions that are sufficiently accurate. As well as automatically defining the density threshold levels, the TPS allows them to be manually defined by the user. Especially important for larger-sized patients, this approach could be used to ensure an appropriate proportion is assigned as lower-density adipose tissue. However, tissue assignment as a manual process is subject to error, which was not investigated in this study but will be explored in future work. Although this study involved a small number of cases in each of the three treatment sites, we believe that we have identified patients with minimal anatomic differences between the CBCT and PCT_CBCT_, enabling the accuracy of CBCT dose calculation to be assessed and for the RS_auto_ method to be validated.

## Conclusion

We investigated various methods, including RS_auto_ that has not been assessed before, for CTN adjustment for CBCT dose calculations. RS_auto_ methods were validated, resulting in CBCT dose calculations for lung patients, for patients treated in the pelvis with D_AP_ < 25 cm, and for H&N cases, which were similar to those calculated on diagnostic CT images. Its implementation into a commercially available TPS makes the RS_auto_ approach the fastest and easiest-to-use of all methods investigated in this study and it can easily be performed as part of a typical clinical workflow. The differences reported in this study between the doses calculated on the CBCT compared with the ground truth, when using density overrides, were similar to other reported results [12] and better than those that use CBCT-density look-up table methods [12]. However, in this study we used a planning CT acquired on the same day as the CBCT as the ground truth in order to minimize the uncertainties often present in studies of this kind. Although the RayStation TPS was used for this work, the methods described could be replicated in other treatment planning systems. Furthermore, the density override methods for CTN adjustment that were investigated in this work are applicable and transferable to dose calculation on segmented MR images.
